# The mediating role of nutritional indicators in the association between estimated glomerular filtration rate and cognitive impairment in older adults

**DOI:** 10.1111/psyg.13176

**Published:** 2024-08-16

**Authors:** Xiuping An, Yao Cui, Mingzhao Qin, Jian Zhou, Qian Liu, Hui Ye

**Affiliations:** ^1^ Department of Geriatrics Beijing Tongren Hospital, Capital Medical University Beijing China

**Keywords:** cognitive impairment, eGFR, mediation, nutrition, older people

## Abstract

**Background:**

Cognitive impairment (CI) is common in older adults, especially those with renal dysfunction. We aimed to investigate the complex relationships among renal function, nutritional status, and CI in older people free from late chronic kidney disease (CKD) and severe CI.

**Methods:**

A study of older people (≥60 years old) with an estimated glomerular filtration rate (eGFR) of >30 mL/min/1.73 m^2^ and Montreal Cognitive Assessment (MoCA) scores of >10 (*n* = 237) was conducted at Beijing Tongren Hospital. Their eGFR was determined using the CKD‐EPI‐cr‐Cysc equation. Cognitive function was evaluated with the MoCA. We tested the relationship between eGFR and MoCA scores using Spearman correlation analysis and multivariate logistic regression analysis. We then conducted a mediation analysis to figure out the mediating roles of nutritional indicators (Mini Nutritional Assessment‐Short Form (MNA‐SF) scores, albumin (ALB), and haemoglobin (HGB)) between the eGFR and MoCA scores.

**Results:**

The incidence of CI was 48.5% (115/237) in older people. Spearman correlation analysis revealed that the better the kidney function, the better the cognitive function (R = 0.297, *P* < 0.001). Multivariate logistic regression analysis revealed that eGFR decrease per 15 mL/min/1.73 m^2^ (OR: 1.415, 95% confidence interval: 1.055–1.896, *P* = 0.020) was related to CI after adjusting for age and sex. However, the eGFR was not associated with cognitive decline after adjusting for nutritional indicators, behavioural risk factors, other biomarkers, and chronic conditions, suggesting that eGFR is not independently associated with CI. Mediation analysis revealed that the MNA‐SF scores (a*b = 0.006 (0.0002–0.012)) and HGB (a*b = 0.008 (0.001–0.017)) were mediating factors between the eGFR and MoCA scores.

**Conclusions:**

A decline in renal function can directly lead to CI and can also exacerbate cognitive deficits through intermediary factors such as MNA‐SF scores and HGB. Therefore, correcting anaemia and improving nutritional status are significantly important for enhancing cognitive function in older patients, especially those with renal dysfunction.

## INTRODUCTION

As the global population ages, the burden of chronic diseases among the older population is becoming increasingly significant. By 2040, chronic kidney disease (CKD) is expected to become the fifth leading cause of death worldwide.^1^ Renal dysfunction and cognitive impairment (CI) are common health concerns among older people. CI, which includes a range of memory, learning, comprehension, and decision‐making deficits, is becoming increasingly prevalent among the older population. Not only does CI affect the daily lives of older adults, but it also increases the risk of chronic illnesses and falls.^2^ Severe cognitive deficits can result in serious consequences such as amnesia, aphasia, disorientation, inability to perform self‐care, or the need for continuous supervision. This imposes a significant burden on families and society. Therefore, early detection and intervention in CI can reduce the occurrence of negative outcomes.

CI is observed in patients at all stages of CKD, including the early stages of the disease, although research in this area is limited.^3^, ^4^ Some studies suggest that cognitive function deteriorates as renal function declines,^5^, ^6^ while others have reported that the relationship between renal function and cognitive function is not simply linear.^7^ These inconsistent findings suggest that there are mediating factors influencing the relationship between CKD and cognitive function.

Malnutrition prevails among individuals with renal dysfunction.^8^, ^9^ At‐risk older adults for malnutrition exhibit a higher propensity for CI.^10^, ^11^ Research has demonstrated that protein consumption in older persons may influence cognitive domains such as memory, visuospatial abilities, language fluency, and attention.^12^ Thus, malnutrition may constitute a significant pathological factor that underlies the link between renal function and the deterioration of cognitive function.^13^ Nevertheless, scant research has been directed toward elucidating the mediating role of malnutrition in the comorbidity of these two conditions. Mild CI, when identified at an incipient stage, may be amenable to reversal with the implementation of suitable early intervention strategies.^14^ Consequently, prompt detection and intervention are of paramount importance.

The objective of this study is to explore the association between reduced renal function and CI, and the mediating role of malnutrition in this relationship in an older population free from late CKD and severe CI. This investigation aims to provide valuable insights for future interventions aimed at enhancing cognitive function in older adults.

## METHODS

### Participants

This study consecutively enrolled 237 older patients who were admitted to the Department of Geriatrics of Beijing Tongren Hospital between 1 January 2020 and 31 March 2023. All enrolled patients provided written informed consent following the Declaration of Helsinki, and the study protocol was approved by the Ethics Committee of Beijing Tongren Hospital, affiliated to Capital Medical University (approval no. TRECKY2021‐042).

The inclusion criteria were as follows: (i) being ≥60 years old, (ii) consenting to participate in the study and undergo relevant tests, and (iii) having no obvious hearing impairment.

The following conditions were criteria for exclusion: (i) complications involving underlying diseases that may affect renal function, such as acute onset of heart, lung, or kidney disease, and so on; (ii) a previous diagnosis of dementia, a Montreal Cognitive Assessment (MoCA) score of ≤10, or the taking of anti‐dementia medications; (iii) complications involving severe neurological diseases which may lead to dementia, such as degenerative diseases of the central nervous system (Alzheimer's disease, Lewy body dementia, Parkinson's disease, Huntington's disease), and non‐degenerative diseases of the central nervous system (vascular dementia such as stroke and cerebral haemorrhage, space‐occupying lesions such as brain tumour, infection such as meningoencephalitis, traumatic brain dementia, hydrocephalus, endocrine and metabolic disorders, poisoning, hypoxia, and paraneoplastic syndrome); (iv) severe neurological or mental illness; (v) vestibular system disease; (vi) the taking of drugs that may lead to cognitive decline or kidney dysfunction; and (vii) an estimated glomerular filtration rate (eGFR) of <30 mL/min/1.73 m^2^.

### Medical history collection and evaluation

#### Clinical data

Basic clinical data of all patients were measured/calculated and recorded, such as sex, age, height, weight, body mass index (BMI), education, blood pressure at admission, smoking and alcohol history, etc. Polypharmacy was defined as the use of five or more drugs. Diagnoses and comorbidities were obtained from medical records, which include any prior health conditions or diseases the patient may have had. Chronic diseases were noted, including CKD, hypertension, diabetes, coronary heart disease, cerebrovascular disease (lacunar infarcts, cerebral artery thrombosis, stenosis, occlusion, cerebral arteritis, cerebral artery injury, cerebral aneurysm, intracranial vascular malformation, cerebral arteriovenous fistula, etc.), and multiple medication history.

#### Laboratory tests

Blood tests were run to determine the levels of participants’ haemoglobin (HGB), serum creatinine (SCr), cystatin C (CysC), uric acid (UA), albumin (ALB), haemoglobin A1C (HbA1C), glucose, triglyceride (TG), total cholesterol (TC), high‐density lipoprotein cholesterol (HDL‐C), and low‐density lipoprotein cholesterol (LDL‐C).

#### Measurement of kidney function

SCr and CysC levels were both measured at admission. The eGFR was calculated from the first SCr value and CysC measurement during hospitalization using epidemiology collaboration (EPI‐cr‐CysC) equations.^15^

#### Comprehensive geriatric assessment

##### Cognitive Assessment—MoCA

The MoCA is a validated brief neuropsychological instrument that effectively screens for CI.^16^ The MoCA Beijing version 7^17^ was administered to all participants in this study. The MoCA has a total score of 30 points, comprised of Simple Task Completion (2 points), Drawing a Clock (3 points), Naming Objects (3 points), Focusing Attention (3 points), Arithmetic Ability (3 points), Language Test (3 points), Abstract Thinking (2 points), Short‐Term Memory Recall Task (5 points), and Orientation in Time and Space (6 points). The MoCA must be completed within 10 min, and the MoCA total score and executive function subscale scores were calculated for each participant.^17^ We used the MoCA cutoff value (<26 indicating CI) in agreement with Nasreddine's study^16^ and referred to the revised Mayo Clinic criteria^18^ to diagnose CI. All patients were divided into a normal group with a MoCA score of ≥26 and a CI group with a MoCA score of <26.

##### Nutritional Assessment

The Mini Nutritional Assessment‐Short Form (MNA‐SF) has been established as a widely utilized tool for evaluating malnutrition risk in the older population.^19^ Lomivorotov *et al*. compared four malnutrition screening tools, and concluded that the MNA‐SF detected a higher number of elderly patients at risk of malnutrition.^20^ The nutritional status of enrolled patients was assessed using the MNA‐SF. The MNA‐SF includes the following six domains: loss of appetite (0–2 points), weight loss (0–3 points), mobility (0–2 points), stress/acute illness (0 or 2 points), neuropsychological impairment (0–2 points), and BMI (0–3 points). If it was not possible to weigh a patient, calf circumference was used as a substitute for BMI (0 or 3 points). According to the total score of the MNA‐SF, patients were classified into three categories: malnourished (0–7 points), risk of malnutrition (8–11 points), and normal nutritional status (12–14 points).^19^

Anaemia and hypoproteinemia are common in old patients with malnutrition. HGB and ALB are routinely measured at admission and thus are easily obtained, which can be a supplement for nutritional status assessment.

##### Evaluation of anxiety and depression

Anxiety and depression were assessed using Zung's Self‐rating Anxiety Scale (SAS) and Self‐rating Depression Scale (SDS)^21^, ^22^ in this study, the SAS and SDS are psychometric instruments used to assess an individual's mental health. The assessment scale consists of two subscales, each containing 20 items, using a 4‐point score, where 1 represents ‘it does not happen or rarely’, 2 represents ‘it happens occasionally’, 3 represents ‘it happens frequently’, and 4 means ‘it happens very frequently or constantly’. The scores on each subscale were aggregated into an overall score, which was then multiplied by 1.25 to produce a standardized score. This tool is valuable in identifying signs of depression in patients.

### Statistical analysis

Data were analysed by using IBM SPSS Statistics for Windows, Version 26.0 (IBM Corp, Armonk, NY, USA), and GraphPad Prism 6 was used for mapping. The Shapiro–Wilk test was used to test the normality assumption of continuous variables. All continuous variables with normal distribution were presented as mean and standard deviation (SD), and non‐normally distributed continuous variables were summarized as median and interquartile range (25th–75th). Intergroup comparisons were performed using an independent *t*‐test or Mann–Whitney *U*‐test when the distribution and variance met the appropriate conditions. Categorical variables were expressed as frequency (percentage) and analysed using the chi‐squared test or Fisher's exact test when needed. Spearman's correlation analysis was used to evaluate the correlation between the eGFR and MoCA score. Variables found to be statistically significant (*P* < 0.05) between the two groups were included in the multivariate logistic regression analysis. Odds ratios (ORs), with 95% confidence intervals and *P*‐values, were reported. Statistical significance was defined as *P* < 0.05.

Finally, the PROCESS macro model 4, version 4.0 in SPSS with bias‐corrected 95% confidence intervals with 5000 bootstrapped samples was used to test the hypothesized mediation models between nutritional indicators, eGFR, and CI. The eGFR was the independent variable (X), the MoCA score was the dependent variable (Y), and nutritional indicators were the mediating variables (Ms). Taking into consideration the current understanding of mediation testing, in the present study, Path a represents the relationship between the eGFR and MoCA scores. Path b represents the relationship between nutritional indicators and MoCA scores when controlling for eGFR. Path c represents the relationship between eGFR and MoCA scores (total effect). Path c′ represents the relationship between the eGFR and MoCA sore when controlling for nutritional indicators (direct effect) (Fig. 3a). In addition, the indirect effect (i.e., mediation) is the product of Paths a and b. Mediation is taken to exist if the confidence interval does not include 0.

## RESULTS

### Baseline characteristics

A total of 237 older patients were included in the present study; 115 (48.5%) were in the CI group and 122 (51.5%) were in the normal group. The median age of the patients was 81 (71.0, 86.0) years, 173 (73.0%) were male, 146 (61.6%) were older than 80 years old, and the median eGFR was 69.0 (57.1, 81.0) mL/min/1.73 m^2^. Compared to the normal group, patients in the CI group were older (*P* < 0.001), had a higher proportion of a history of hypertension (*P* = 0.017), cerebrovascular disease (*P* = 0.017), and multiple medication history (*P* = 0.033). However, no statistically significant differences were observed for sex, BMI, smoking history, alcohol history, diabetes, or coronary heart disease between the two groups (*P* > 0.05) (Table 1).

**Table 1:** Baseline characteristics and laboratory indicators of the older adults in the two groups

Variables	All group (*n* = 237)	Normal group (*n* = 122)	CI group (*n* = 115)	|X^2^/Z|	*P*
Age (years)	81.0 (71.0, 86.0)	77.5 (68.8, 84.0)	84.0 (76.0, 87.0)	4.254	<0.001*
Male (*n*, %)	173 (73.0)	91 (74.6)	82 (71.3)	0.324	0.569
BMI (kg/m^2^)	24.0 (22.1, 25.8)	24.0 (22.0, 25.7)	24.1 (22.2, 26.0)	0.446	0.655
Smoking history (*n*, %)	56 (29.9)	28 (29.5)	28 (30.4)	0.021	0.886
Alcohol history (*n*, %)	31 (17.1)	18 (18.9)	14 (15.2)	0.458	0.498
Chronic kidney disease (*n*, %)	17 (7.2)	6 (4.9)	11 (9.6)	1.973	0.160
Hypertension (*n*, %)	167 (70.8)	78 (63.9)	89 (78.1)	5.692	0.017*
Diabetes (*n*, %)	103 (43.6)	51 (41.8)	52 (45.6)	0.348	0.555
Coronary heart disease (*n*, %)	102 (43.2)	49 (40.2)	53 (46.5)	0.961	0.327
Cerebrovascular disease (*n*, %)	47 (19.9)	17 (13.9)	30 (26.3)	5.664	0.017*
Multiple medication history (*n*, %)	119 (50.4)	52 (42.6)	67 (58.8)	6.148	0.013*
SCr (μmol/L)	79.8 (68.0, 95.6)	77.9 (68.0, 91.8)	84.8 (68.0, 99.4)	1.524	0.127
CysC (mg/L)	1.1 (1.0, 1.3)	1.1 (0.9, 1.2)	1.2 (1.0, 1.4)	3.12	0.002*
eGFR (mL/min/1.73 m^2^)	69.0 (57.1, 81.0)	75.1 (63.8, 84.9)	65.0 (52.0, 72.0)	4.785	<0.001*
UA (μmol/L)	346.4 (287.2, 407.8)	333.1 (283.6, 406.3)	360.9 (290.3, 411.6)	0.984	0.325
HGB (g/L)	137.0 (127.0, 144.0)	138.0 (130.0, 145.0)	131.5 (120.0, 142.8)	3.159	0.002*
ALB (g/L)	40.4 (37.9, 43.3)	41.1 (38.5, 43.4)	40.0 (37.4, 43.1)	2.063	0.039*
HbA1C (%)	6.5 (5.8, 7.6)	6.2 (5.6, 7.5)	6.5 (6.0, 7.8)	1.927	0.054
TG (mmol/L)	1.2 (0.8, 1.7)	1.2 (0.8, 1.7)	1.3 (0.8, 1.7)	0.114	0.910
TC (mmol/L)	4.2 (3.6, 4.9)	4.1 (3.6, 4.9)	4.3 (3.5, 4.8)	0.529	0.597
HDL‐C (mmol/L)	1.3 (1.0, 1.7)	1.3 (1.0, 1.7)	1.3 (1.1, 1.7)	0.821	0.411
LDL‐C (mmol/L)	2.2 (1.7, 2.8)	2.3 (1.8, 2.8)	2.2 (1.7, 2.8)	0.211	0.833
MoCA score	26.0 (23.0, 28.0)	28.0 (27.0, 29.0)	23.0 (21.0, 24.0)	13.360	<0.001*
MNA‐SF score	12.0 (11.0, 14.0)	14.0 (11.0, 14.0)	12.0 (10.0, 14.0)	3.003	0.003*
SAS score	32.5 (28.8, 37.5)	31.3 (27.5, 37.5)	32.5 (30.0, 37.5)	1.686	0.092
SDS score	32.5 (28.8, 38.8)	31.3 (27.5, 39.1)	33.8 (28.8, 38.8)	1.603	0.109

*Note*: Continuous variables were summarized as median (25th–75th). Othe values are presented as number (%).

Abbreviations: CI, cognitive impairment; BMI, body mass index; Scr, serum creatinine; CysC, cystatin C; eGFR, estimated glomerular filtration rate; UA, uric acid; HGB, haemoglobin; ALB, albumin; HbA1c, glycosylated haemoglobin; TC, total cholesterol; TG, triglyceride; HDL‐C, high‐density lipoprotein cholesterol; LDL‐C, low‐density lipoprotein cholesterol; MoCA, Montreal Cognitive Assessment; MNA‐SF, Mini Nutritional Assessment Short Form; SAS, Self‐rating Anxiety Scale; SDS, Self‐rating Depression Scale.

* *P* < 0.05.

### Laboratory and assessment indicators

There was no statistical difference in SCr between the two groups. However, the eGFR on admission of patients in the CI group was significantly lower than that of the normal group (65.0 (52.0, 72.0) vs 75.1 (63.8, 84.9) mL/min/1.73 m^2^, *P* < 0.001). The HGB (131.5 (120.0, 142.8) vs 138.0 (130.0, 145.0) g/L, *P* = 0.002) and ALB (40.0 (37.4, 43.1) vs 41.1 (38.5, 43.4) g/L, *P* = 0.039) on admission in the CI group were also lower than that of the normal group. There were no significant differences in blood lipid, blood glucose, UA, or HbA1c between the two groups (*P* > 0.05) (Table 1).

The MNA‐SF scores of the CI group were lower than that of the normal group, while the SAS scores and SDS scores in the CI group were not statistically significant between the two groups (*P* > 0.05) (Table 1).

### Correlation between eGFR and MoCA scores (Spearman correlation analysis)

**Figure 1:**
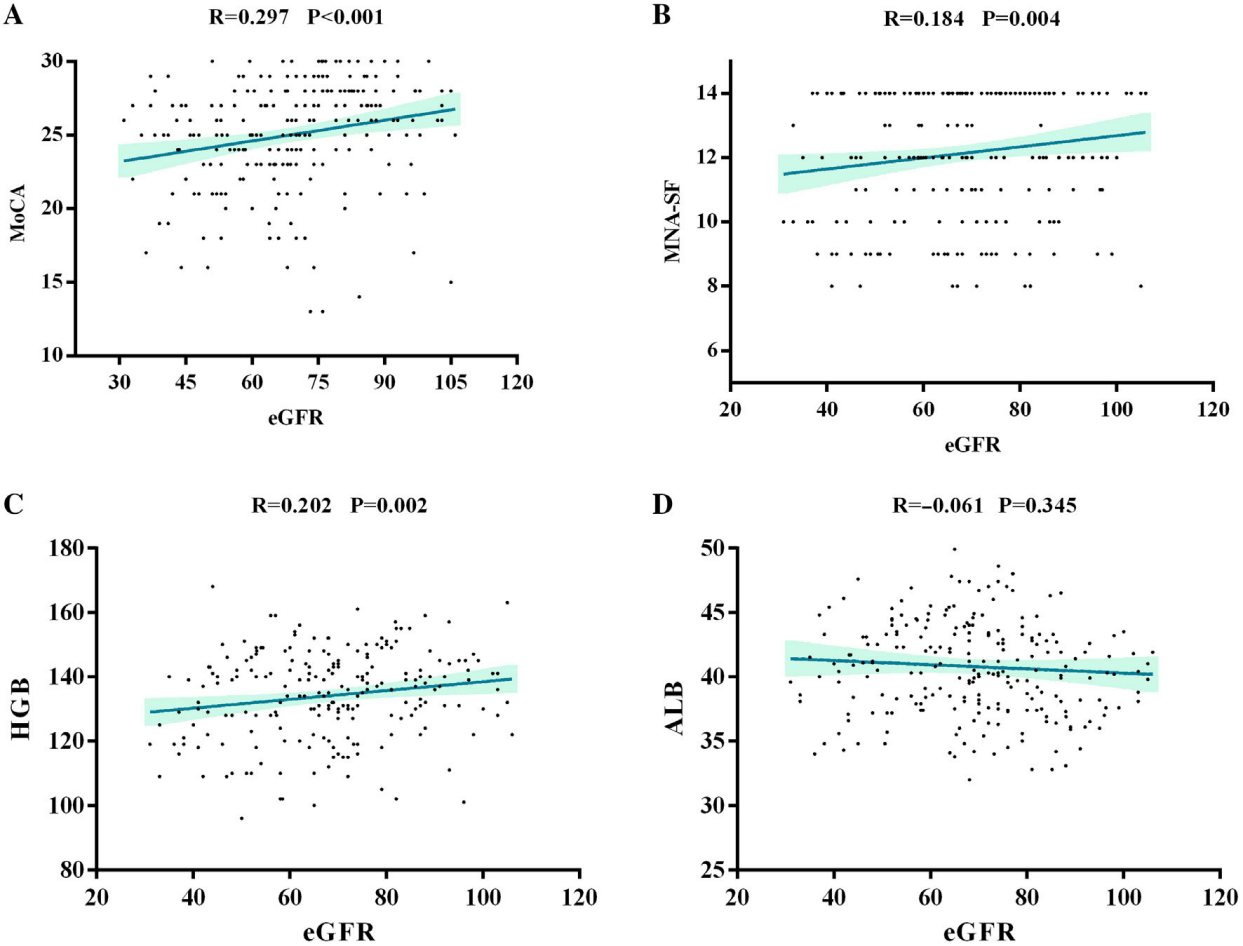
Spearman correlation analysis of eGFR with MoCA scores and nutritional indicators, shown by point and line graph representations of the strength and direction of the correlations. The upper and lower blue lines denote the upper and lower bounds, respectively, of the confidence interval for the correlation coefficient, whereas the central line illustrates the line of best fit for the correlation. (A) eGFR and MoCA scores. (b) eGFR and MNA‐SF scores. (c) eGFR and HGB. (d) eGFR and ALB. ALB, albumin; eGFR, estimated glomerular filtration rate; HGB, haemoglobin; MNA‐SF, Mini Nutritional Assessment‐Short Form; MoCA, Montreal Cognitive Assessment.

Significant trends of lower cognition were observed across the groups for lower eGFR values. The relationship between the eGFR and MoCA score was noted. The better the kidney function, the better the cognitive function (R = 0.297, *P* < 0.001). Detailed results are presented in Fig. 1A.

### Correlation between eGFR and nutritional indicators (Spearman correlation analysis)

The relationship between the eGFR and nutritional indicators (including MNA‐SF scores and HGB) was noted. The better the kidney function, the higher the MNA‐SF scores and HGB (MNA‐SF scores: R = 0.184, *P* = 0.004; HGB: R = 0.202, *P* = 0.002). However, no significant trend was observed between the eGFR and ALB. Detailed results are presented in Fig. 1B–D.

### Correlation between eGFR and MoCA scores (logistic analysis)

**Figure 2:**
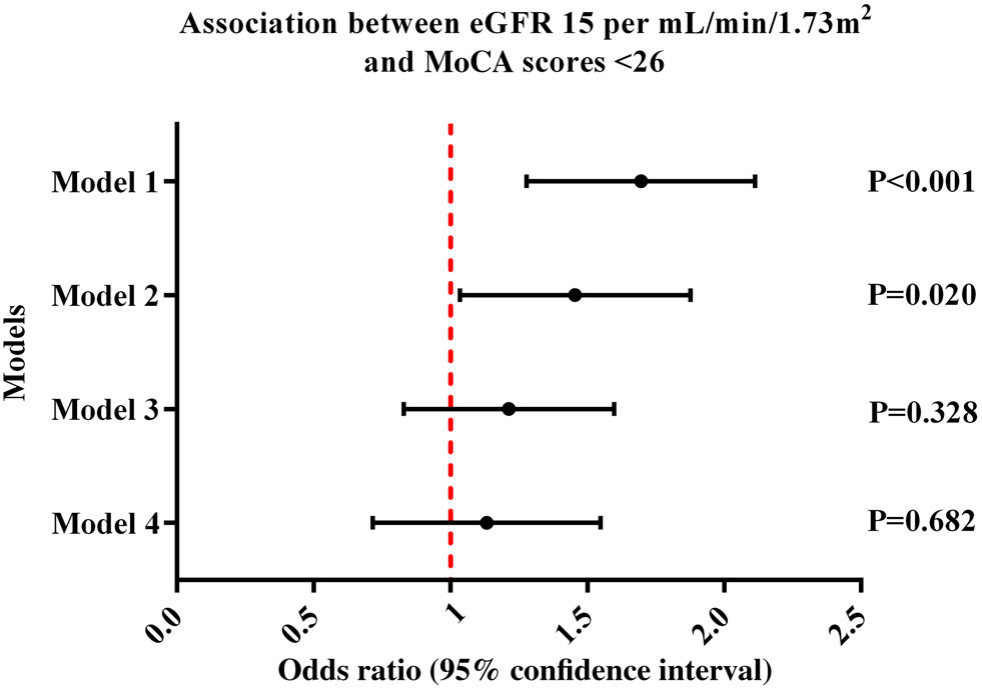
Logistic regression models illustrating the association between eGFR decrease per 15 mL/min/1.73 m^2^ and MoCA scores of <26. Model 1 is unadjusted. Model 2 (demographic) is adjusted for age and sex. Model 3 (nutritional indicators) is adjusted for demographics, MNA‐SF scores, HGB, and ALB. Model 4 (behavioural factors) is adjusted for nutritional indicators, smoking history, alcohol history, BMI, SDS sores, and chronic conditions (hypertension, diabetes, coronary heart disease, cerebrovascular disease). ALB, albumin; BMI, body mass index; eGFR, estimated glomerular filtration rate; HGB, haemoglobin; MNA‐SF, Mini Nutritional Assessment‐Short Form; MoCA, Montreal Cognitive Assessment; SDS, Self‐rating Depression Scale.

Multiple clinical factors contributed to the progression of CI. Logistic regression was used to analyse the correlation between the eGFR and CI (Fig. 2). Converting the eGFR into categorically independent variables at intervals of 15 mL/min/1.73 m^2^, and conducting binary logistic regression with CI (MoCA < 26) as the dependent variable, led to the development of four separate regression models.

We found that per 15 mL/min/1.73m^2^ decrease in the eGFR, the risk of CI increased by 0.662 times (OR = 1.662, 95% confidence interval: 1.297–2.129, *P* < 0.001), as shown in Model 1. After adjusting for age and sex, per 15 mL/min/1.73m^2^ increase in eGFR, the risk of CI increased by 0.415 times (OR = 1.415, 95% confidence interval: 1.055–1.896, *P* = 0.020) as shown in Model 2. However, after adjusting for demographic and nutritional indicators (including MNA‐SF scores, HGB, and ALB), the association between the eGFR and the MoCA scores was not statistically significant (OR = 1.173, 95% confidence interval: 0.852–1.616, *P* = 0.328) as shown in Model 3. Furthermore, after adjusting for demographic and nutritional indicators, smoking and alcohol history, BMI, SDS scores, and chronic conditions (hypertension, diabetes, coronary heart disease, cerebrovascular disease), the OR is closer to 1 (OR = 1.081, 95% confidence interval: 0.744–1.571, *P* = 0.682) as shown in Model 4.

### The mediating effect of nutritional indicators between the eGFR and MoCA scores

**Figure 3:**
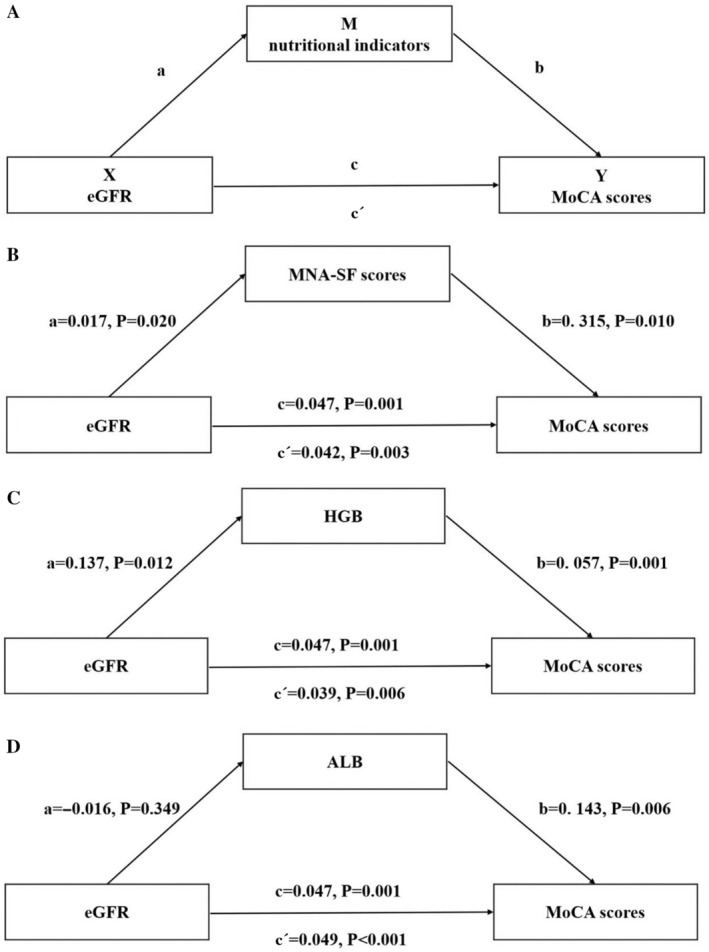
(A) Schematic diagram of the mediation effect model. (B) Mediating effect of MNA‐SF scores between the eGFR and cognitive function. (C) Mediating effect of HGB between the eGFR and cognitive function. (D) Mediating effect of ALB between the eGFR and cognitive function. ALB, albumin; eGFR, estimated glomerular filtration rate; HGB, haemoglobin; MNA‐SF, Mini Nutritional Assessment‐Short Form; MoCA, Montreal Cognitive Assessment.

The mediation models showed that the eGFR had a significant effect on MNA‐SF scores (a = 0.017, *P* = 0.020) and HGB (a = 0.137, *P* = 0.012), but no correlation was found between eGFR and ALB (a = −0.016, *P* = 0.349). The eGFR can also predict MoCA scores (*P* < 0.05). MNA‐SF scores (b = 0.315, *P* = 0.010), ALB (b = 0.143, *P* = 0.006), and HGB (b = 0.057, *P* = 0.001) were both significant predictors of MoCA scores. The bias‐corrected percentile bootstrap method was used to estimate the interval of the mediation effect, and the bootstrap number was set to 5000. The results showed that the confidence interval of the effect of the eGFR on MoCA scores did not contain 0 (Fig. 3), indicating that the direct effect was significant. The confidence intervals for the indirect effect of MNA‐SF scores and HGB also do not contain 0, indicating that the mediation effect is significant, and the relative mediation effect is 11.7% and 16.4%, respectively (Table 2, Fig. 3A,B) However, the confidence interval for the indirect effect of ALB (Table 2, Fig. 3C) contains 0, indicating that the mediation effect is not significant. These results indicate that renal function can not only directly affect cognition, but also indirectly lead to cognitive decline through malnutrition and anaemia.

**Table 2:** The mediating effect of nutritional indicators between the eGFR and MoCA scores

eGFR→nutritional indicators→MoCA	c	c′	a	b	a*b	a*b 95% Boot CI	Effect ratio
eGFR→MNA‐SF → MoCA	0.047	0.042	0.017	0.315	0.006	0.0002–0.012	11.7%
eGFR→ALB→MoCA	0.047	0.049	−0.016	0.143	−0.002	−0.008–0.002	No mediating effect
eGFR→HGB → MoCA	0.047	0.039	0.137	0.057	0.008	0.001–0.017	16.4%

*Note*: Paths in the mediation analysis are represented as follows: c = the regression coefficient of the independent variable to the dependent variable, i.e. the total effect;

c′ = the regression coefficient of the independent variable to the dependent variable, i.e. the direct effect;

a = the regression coefficient of the independent variable to the mediating variable;

b = the regression coefficient of the mediating variable to the dependent variable;

a*b = the mediating effect.

The 95% confidence interval calculated by bootstrap sampling is indicated as 95% Boot CI. If the interval does not include 0, it indicates a mediating effect.

Abbreviations: eGFR, estimated glomerular filtration rate; MoCA, Montreal Cognitive Assessment; MNA‐SF, Mini Nutritional Assessment Short Form; ALB, albumin; HGB, haemoglobin.

## DISCUSSION

Globally, up to 10% of adults suffer from CKD, a condition that is often irreversible and usually progressive. The prevalence of renal dysfunction has significantly increased, especially among older adults.^23^ Patients with renal dysfunction are at a significantly higher risk of developing CI compared to the general population.^24^ CI is increasingly recognized as a significant factor in chronic disability,^25^ highlighting the importance of early detection and preventive measures. Current research on the relationship between the eGFR and cognitive function has mainly concentrated on patients with end‐stage renal disease. There is a lack of studies investigating the connection between CI and less severe forms of kidney disease. This study revealed that older adults free from late CKD and severe CI in the CI group showed a higher prevalence of hypertension and cerebrovascular disease. This corroborates previous findings that identify these conditions as crucial risk factors for vascular CI and dementia in later life.^26^ Additionally, the present study found that levels of CysC were significantly higher in the CI group compared to the normal group (*P* = 0.002), and the eGFR was also found to be lower in the CI group (*P* < 0.001). However, the creatinine is not different between the two groups. We thought the reason is that creatinine is affected by muscle mass, which in turn changes with older age, and many other factors such as dietary protein intake, malnutrition, and prescribed medication can also affect the production and secretion of creatinine in older people. Furthermore, the levels of HGB (*P* = 0.002) and ALB (*P* = 0.039) were also reduced compared to the normal group in this study.

Prior longitudinal studies had identified renal dysfunction as one of the most potent risk factors for CI and dementia, ranking second only to stroke and prolonged use of anti‐anxiety medications.^24^ A multitude of research has suggested that both reduced eGFR and proteinuria are associated with a decline in cognitive function.^5^ In the Spearman correlation analysis, the eGFR showed a positive correlation with MoCA scores. When using MoCA scores as the dependent variable and eGFR as the independent variable in univariate regression analysis, a significant association was observed between eGFR and MoCA scores, as indicated in Model 1. This association persisted even after adjusting for age and sex (as outlined in Model 2), suggesting that a decline in renal function is accompanied by a deterioration in cognitive function. The progression of renal impairment could be a factor that can be modified to reduce the risk of cognitive decline and its progression. However, after controlling for demographic factors (including age and sex) and nutritional indicators (including MNA‐SF scores, HGB, and ALB) in the multiple logistic regression analysis, the eGFR did not maintain an independent association with CI. This indicates an interaction between nutritional indicators and the relationship between eGFR and MoCA scores, as shown in Model 3. Moreover, in Model 4, which adjusts for lifestyle (including smoking history, alcohol history, and BMI) and chronic conditions (hypertension, diabetes, coronary heart disease, cerebrovascular disease) that may influence CI, the OR for eGFR approached 1. This means some other clinical factors such as lifestyle may interact with the relationship between the eGFR and MoCA scores. These findings suggest that the eGFR is not an independent risk factor for CI in older patients. A comprehensive study involving two nationally representative cohorts of aging individuals reported that a decline in eGFR‐CysC is significantly linked to accelerated cognitive decline after extensive adjustments, independently of SCr/eGFR‐creatinine (eGFR‐cr).^27^ In a prospective observational study, elevated levels of CysC were associated with lower cognitive performance after adjusting for age, race, education, and medical comorbidities in linear models. This association remained statistically significant even after further adjustment for eGFR.^28^ However, in a longitudinal analysis of a prospective cohort consisting of 1332 older women without dementia, the association between CysC levels and CI or dementia was not found to be independent of potential confounding factors.^29^ These findings collectively suggest that CysC is associated with cognitive function, and the presence of mediating factors is also implicated in this relationship.

In this study, mediating effects analysis showed that the eGFR has a direct effect on cognitive decline, and MNA‐SF scores and HGB have a partial mediating effect on the relationship between renal function and cognitive decline. In other words, the lower the eGFR, the worse the nutritional status, and the worse the cognitive level. Unfortunately, we could not detect a mediating effect of ALB on renal function and CI. Since there is a lack of relevant studies reporting the mediating effects of nutritional indicators on eGFR and cognitive decline in older patients, studies at different levels provide indirect evidence. Patients with renal dysfunction are highly susceptible to malnutrition^30^ and anaemia.^31^ The aetiology of these conditions includes reduced erythropoietin synthesis, decreased red blood cell survival, platelet dysfunction leading to haemorrhage, and gastrointestinal malabsorption resulting in severe iron deficiency.^32^ Furthermore, older individuals with renal dysfunction are more susceptible to malnutrition due to factors such as reduced appetite, hormonal metabolic dysregulation, inflammatory responses, and increased catabolism.^33^ Nutritional status has also been associated with cognitive function. Numerous studies have indicated that nutritional supplementation can lead to promising outcomes for cognitive health.^34^, ^35^ Specific deficiencies in nutrients may worsen the physiological mechanisms in the brain that increase susceptibility to DNA damage in neuronal cells, leading to cognitive decline.^36^ Mechanistic studies have shown that proteins and amino acids are essential for maintaining neuronal integrity, reducing inflammation, and preserving muscle mass.^37^

Our findings emphasize the importance of considering nutritional status as a potential factor that can be modified in the management of cognitive decline among older patients, especially those with CKD. While the mediating role of ALB in the relationship between renal function and CI remains unclear in our study, the established associations between eGFR, MNA‐SF scores, HGB, and cognitive outcomes suggest that interventions aimed at improving nutritional status and addressing anaemia may have a beneficial impact on cognitive health. Given the limited research specifically addressing the mediating effects of nutritional indicators on the relationship between eGFR and cognitive decline in this population, our study provides valuable insights and calls for further investigation in this area. Furthermore, for older people, especially those with kidney dysfunction, it is crucial to integrate thorough nutritional assessments into regular clinical practice to identify those at high risk of cognitive decline. For those at greater risk, it is vitally important to implement early preventive measures to protect cognitive function. First, high‐risk groups should avoid taking nephrotoxic drugs, and adjust their diet and medications to slow the progression of decline in kidney function. Besides, patients with anaemia should supplement raw materials for blood. Furthermore, in those cases, targeted supplementation of macro‐ and/or micronutrients and diet to improve nutritional status may be beneficial for cognition.

There were a few limitations to our study. First, this was a single‐centre study with a small sample size; therefore, selection bias and confounding bias were unavoidable, which requires a multi‐centre, large‐sample study. Besides, this was a cross‐sectional observational study, and as such, a causal association cannot be established, and prospective studies should further confirm the conclusions. Future research should focus on longitudinal studies to clarify the causal relationships and investigate the potential for nutritional interventions to act as a protective strategy against cognitive decline in older people. Moreover, there may be other mediating factors between the eGFR and cognition, but this study only explored the MNA‐SF scores, ALB, and HGB; thus future research could focus on the intermediation of other clinical factors.

## CONCLUSION

This study investigated the intricate relationship between renal function, nutritional status, and CI in older patients. The results indicate that a decline in renal function can directly lead to CI and can also worsen cognitive deficits through intermediary factors such as malnutrition and anaemia. Therefore, correcting anaemia and improving nutritional status are significantly important in enhancing cognitive function in older individuals, especially those with renal dysfunction. Incorporating the assessment of nutritional indicators into the routine care of older adults may be crucial for the early identification of CI and the improvement of cognitive health.

## ETHICS STATEMENT

This project complies with the current laws and ethical standards of the country in which it was performed. This study was conducted in accordance with the Declaration of Helsinki. This study complied with the requirements of medical ethics and was reviewed and approved by the ethics committee of Beijing Tongren Hospital Affiliated to Capital Medical University (approval No. TRECKY2021‐042). All participants were informed of the purpose of this study and gave signed written informed consent.

## AUTHOR CONTRIBUTIONS

AXP and CY conceptualized and designed the study, drafted the initial manuscript, and reviewed and revised the manuscript. AXP, CY, ZJ, LQ, and YH designed the data collection instruments, collected data, carried out the initial analyses, and reviewed and revised the manuscript. CY, QMZ coordinated and supervised data collection, and critically reviewed the manuscript for important intellectual content. All authors approved the final manuscript as submitted and agreed to be accountable for all aspects of the work.

## CONSENT FOR PUBLICATION

Not applicable.

## Data Availability

The data that support the findings of this study are available from the corresponding author upon reasonable request.
